# Anxiety- and depression-like behavior in mice lacking the *CD157/BST1* gene, a risk factor for Parkinson's disease

**DOI:** 10.3389/fnbeh.2014.00133

**Published:** 2014-04-22

**Authors:** Olga Lopatina, Toru Yoshihara, Tomoko Nishimura, Jing Zhong, Shirin Akther, Azam A. K. M. Fakhrul, Mingkun Liang, Chiharu Higashida, Kohei Sumi, Kazumi Furuhara, Yuki Inahata, Jian-Jung Huang, Keita Koizumi, Shigeru Yokoyama, Takahiro Tsuji, Yulia Petugina, Andrei Sumarokov, Alla B. Salmina, Koji Hashida, Yasuko Kitao, Osamu Hori, Masahide Asano, Yoji Kitamura, Takashi Kozaka, Kazuhiro Shiba, Fangfang Zhong, Min-Jue Xie, Makoto Sato, Katsuhiko Ishihara, Haruhiro Higashida

**Affiliations:** ^1^Department of Basic Research on Social Recognition and Memory, Research Center for Child Mental Development, Kanazawa UniversityKanazawa, Japan; ^2^Core Research for Evolutional Science and TechnologyTokyo, Japan; ^3^Department of Biochemistry, Medical, Pharmaceutical and Toxicological Chemistry, Krasnoyarsk State Medical UniversityKrasnoyarsk, Russia; ^4^Advanced Science Research Center, Kanazawa UniversityKanazawa, Japan; ^5^Department of Neuroanatomy, Kanazawa University Graduate School of Medical SciencesKanazawa, Japan; ^6^Division of Cell Biology and Neuroscience, Department of Morphological and Physiological Sciences, Faculty of Medical Sciences, University of FukuiFukui, Japan; ^7^Research Center for Child Mental Development, University of FukuiFukui, Japan; ^8^Department of Immunology and Molecular Genetics, Kawasaki Medical SchoolKurashiki, Japan

**Keywords:** BST-1, emotion-related behavior, social behavior, oxytocin, non-motor symptoms

## Abstract

CD157, known as bone marrow stromal cell antigen-1, is a glycosylphosphatidylinositol-anchored ADP-ribosyl cyclase that supports the survival and function of B-lymphocytes and hematopoietic or intestinal stem cells. Although *CD157/Bst1* is a risk locus in Parkinson's disease (PD), little is known about the function of CD157 in the nervous system and contribution to PD progression. Here, we show that no apparent motor dysfunction was observed in young knockout (*CD157*^−/−^) male mice under less aging-related effects on behaviors. *CD157*^−/−^ mice exhibited anxiety-related and depression-like behaviors compared with wild-type mice. These behaviors were rescued through treatment with anti-psychiatric drugs and oxytocin. CD157 was weakly expressed in the amygdala and c-Fos immunoreactivity in the amygdala was less evident in *CD157*^−/−^ mice than in wild-type mice. These results demonstrate for the first time that CD157 plays a role as a neuro-regulator and suggest a potential role in pre-motor symptoms in PD.

## Introduction

Parkinson's disease (PD) is considered to be a movement disorder, and its diagnosis is based on the presence of a set of cardinal motor symptoms (Samil et al., 2004; Jankovic, 2008). Recently, considerable evidence has shown that the neurodegenerative processes that lead to sporadic PD begin many years before the appearance of the characteristic motor symptoms associated with nigro-striatal dopaminergic neuron loss (Leentjens et al., 2011; Noyce et al., 2012; Prediger et al., 2012). Thus, abnormalities in additional neuronal regions, including the amygdala, are potentially involved in PD progression and may contribute to the appearance of non-motor symptoms (Tessitore et al., 2002; Hälbig et al., 2011), such as anxiety, depression, olfactory and memory impairment, sleep abnormalities and gastrointestinal disturbances (Menza et al., 1993; Chaudhuri et al., 2006; Reijnders et al., 2008; Chaudhuri and Schapira, 2009). Anxiety and depression are the earliest PD manifestations, and patients with high anxiety are at an increased risk for PD (Frisina et al., 2009; Bower et al., 2010; Nègre-Pagès et al., 2010). The underlying biological mechanisms that lead to these symptoms during any stage of the disease, including the pre-motor phase, are unknown (Richard, 2005; Tadaiesky et al., 2008; Siderowf and Lang, 2012; Pontone et al., 2013).

Interestingly, genome-wide association studies and meta-analysis for PD have identified intronic single-nucleotide polymorphisms (SNPs) in the *CD157/BST1* gene on human chromosome 4p15 as new susceptibility loci in several populations (Satake et al., 2009; Tan et al., 2010; Liu et al., 2011, 2013b; Nalls et al., 2011; Saad et al., 2011; Simón-Sánchez et al., 2011; Spencer et al., 2011; Zimprich, 2011; Lill et al., 2012; Sharma et al., 2012), with reported variations (Chang et al., 2011; Miyake et al., 2012; Wang et al., 2012; Zhu et al., 2012; Chen et al., 2013; Chung et al., 2013). These studies indicate that CD157 might be associated with the causality of PD, or at least one of a variety of PD symptoms.

Bone marrow stromal cell antigen-1 (BST-1) was first isolated as a cell surface molecule that supports the pre-B cell growth with enhanced expression on the bone marrow stromal cell lines derived from rheumatoid arthritis patients (Kaisho et al., 1994; Ishihara and Hirano, 2000). BST-1 was also expressed on myeloid cells as a molecule capable of signal transduction and clustered in CD157 in Leucocyte Typing IV after genetic cloning (Itoh et al., 1994; Muraoka et al., 1996; Okuyama et al., 1996; Ishihara et al., 1997). CD157/BST-1 is a member of the NADase/ADP-ribosyl cyclase family, which includes CD38 (Hirata et al., 1994; Itoh et al., 1994; Ferrero et al., 1999; Ishihara and Hirano, 2000; Guse, 2005; Malavasi et al., 2006, 2008; Lee, 2012; Quarona et al., 2013). CD157 plays a variety of roles in humoral immune responses, neutrophil transmigration and haematopoietic stem cell support (Ishihara and Hirano, 2000; Funaro et al., 2004; Podestà et al., 2005; Malavasi et al., 2006; Mouchiroud et al., 2013). CD157 is also involved in the pathophysiology of various diseases, such as the survival of B cells in rheumatoid arthritis, the progression of leukaemia and metastasis of human ovarian carcinoma cells (Kaisho et al., 1994; Shimaoka et al., 1998; Ishihara and Hirano, 2000; Ortolan et al., 2010; Quarona et al., 2013; Lo Buono et al., 2014). In addition, a new role for CD157 has been reported in stem cells: CD157 induces the catalysis of cyclic ADP-ribose in paneth cells, which promotes intestinal stem cell self-renewal in mice on a calorie-restricted diet (Yilmaz et al., 2012); or lung stem/progenitor cells express CD157 (Wu et al., 2013). However, these reports revealed little or no information concerning the relationship with brain function or brain deficits in PD.

In this study, to clarify the neuronal functions of CD157/BST-1, we attempted to characterize the motor and social behaviors in young adult (8- to 10-week-old) male *CD157* knockout (*CD157*^−/−^) mice (Itoh et al., 1998), specifically while controlling for any aging-related effects on behaviors. We show that *CD157*^−/−^ mice exhibit no motor dysfunction, but severe anxiety-related and depression-like behaviors, which were reversed through drug treatment.

## Materials and methods

### Animals

*CD157/Bst1* knockout (*CD157*^−/−^) mice were previously described (Itoh et al., 1998). Frozen *CD157*^+/−^ fertilized oocytes were routinely inoculated into psuedopregnant foster mothers. Offspring were genotyped as previously described (Itoh et al., 1998). *CD157*^−/−^ mice were maintained by crossbreeding homozygous mutant mice. C57BL/6 wild-type (*CD157*^+/+^) and CD157^−/−^ mice were maintained at the animal center under standard conditions (22°C; 12-h light/dark cycle, lights on at 8:45 AM) in standard mouse cages (300 × 160 × 110 mm) with sawdust bedding and food and water *ad libitum*. The breeding pairs were maintained separately (1 pair per cage). After parturition, pup body weight of both males and females was measured daily in 10 pairs containing approximately 40 pups. At 21 days old, the offspring were removed for weaning and housed in same-sex sibling pairs. Subsequently, viability was assessed. All animal experiments were carried out in accordance with the Fundamental Guidelines for Proper Conduct of Animal Experiment and Related Activities in Academic Research Institutions under the jurisdiction of the Ministry of Education, Culture, Sports, Science and Technology of Japan, and were approved by the Committee on Animal Experimentation of Kanazawa University.

### Rabbit anti-CD157 antibody

An antibody that specifically recognizes murine CD157 (BST-1) was used in this study. Anti-murine CD157 antiserum was prepared by immunizing rabbits with a chimeric fusion protein of murine CD157 and the Fc portion of human IgG1 (mBST-1Fc), and the reactivities to human IgG and murine CD38 were absorbed by human IgG sepharose 6 and the transfectant, BAFmCD38, respectively (Kajimoto et al., 1996). In some experiments involving histochemical staining in the brain, we also used CD157 mAb (clone BP-3; Becton Dickinson, NJ, USA). We obtained identical staining results from both antibodies.

Adult mice were sacrificed through transcardial perfusion with 4.0% paraformaldehyde in phosphate buffered saline (PBS, Gibco, Life Technologies, Tokyo). Subsequently, the spleen was removed, fixed overnight and cryoprotected in PBS containing 15 and 30% sucrose. The spleen was cut into 20-μ m-thick sections using a freezing microtome. The sections were washed in PBS to wash out the OCT compound and permeabilised with 0.3% TritonX-100 in PBS for 30 min. Subsequently, the sections were blocked in PBS containing 0.3% TritonX-100 and 3% BSA (bovine albumin, F-V, pH 5.2; Nacalai Tesque, Kyoto, Japan) for 1 h at room temperature and incubated for 2 nights at 4°C with primary anti-murine CD157 antiserum (1:100). The sections were washed with 0.3% TritonX-100 in PBS and incubated with goat anti-rabbit IgG Alexa Fluor 488 (Invitrogen Molecular Probes; Tokyo, Japan; 1:200) and DAPI (Dojindo, Kumamoto, Japan; 1:2000) for 1 h at room temperature. Imaging was performed with an Olympus IX71 fluorescence microscope (Tokyo, Japan).

### Footprint pattern analysis

The footprint test was used to compare the gaits of *CD157*^+/+^ and *CD157*^−/−^ mice (Steele et al., 2007). Hindfeet and forefeet were coated with a black non-toxic paint, and the animals were allowed to walk along a 50-cm-long, 10-cm-wide runway (with 15-cm high walls) over a fresh sheet of white paper (Nóbrega et al., 2013). The distance between the center of the hindfoot print and the center of the preceding forefoot print was recorded over a sequence of 4 consecutive steps, excluding footprints made at the beginning and end of the run. Two operators made all footprint recordings for 20 mice.

### Rota rod test

To investigate the motor coordination/motor learning abilities, the accelerating rota rod paradigm was used (Yoshihara et al., 2009; Ito et al., 2012). The mice were tested in 3 trials per day for 3 consecutive days with a 300-sec accelerating programme (from 5 to 40 rpm). The latency to fall from the rod was calculated.

### Homecage activity

Mouse spontaneous activity in a familiar environment was recorded using a homecage activity monitoring system (The SmartCage system, O'Hara & Co., LTD. Tokyo, Japan). A 12-h light/dark cycle and LED illumination were programmed (08:45–20:45, light period; 20:45–08:45, dark period). Each mouse was separately housed in a cage, and the mice could access water and food pellets *ad libitum*. The floor size of each homecage was 17 × 25 cm and was illuminated at 50 lux in the light period. Mouse movement was recorded for 24 h (Steele et al., 2007).

### Open field test

The open field test measures locomotion and anxious behaviors (Silverman et al., 2013). The open field box consisted of a square box (600 × 600 × 200 mm), covered with polypropylene sheets inside the wooden box. The center arena (300 × 300 mm) was outlined. Each animal was placed in the box for 10 min. Overall activity in the box was measured, and the amount of time and distance traveled in the center arena was noted. The distance traveled in the field was recorded using a digital video system and ANY-maze software (Stoelting Co., Wood Dale, IL, USA). This paradigm is based on the idea that mice will naturally prefer to be near a protective wall rather than exposed to danger out in the open. After each trial, the test chambers were cleaned with a damp towel and 1% sodium hypochlorite, followed by 70% alcohol (Liu et al., 2013a).

### Social preference task in the open field

This test was performed in the open field chamber. A mouse was first placed in the open field for 10 min (habituation session). After habituation, the mouse was returned to its home cage, and an inanimate object was placed in the center of the field. In the next test, session 1, the mouse was again placed in the open field chamber with the novel non-social object (a wire cage) for next 10 min. Subsequently, the non-social object was changed for a C56BL/6 naïve male mouse under a new wire cage (70 × 90 × 70 mm and bars spaced 5 mm apart). The test mouse was again introduced to the arena for 10 min. The percentage of time spent and the number of entries into the inside zone were analyzed using the digital video system and the ANY-maze video tracking software. At the end of each test, the open field box and inanimate object were sprayed with 1% sodium hypochlorite and subsequently 70% ethanol and were wiped clean with paper towels. The mean time interval between sessions was 2–3 min.

### Sociability and preference for social novelity

The social behavior test was performed using a three-chamber box to assess whether subject mice tend to spend time with stranger mice. The apparatus was a rectangular, three-chambered box covered with clear polycarbonate. Dividing walls had doorways allowing access into each chamber. At the end of each test, the apparatus was sprayed with 1% sodium hypochlorite and 70% ethanol and wiped clean with paper towels. The following procedure was used for the social behavior test: (A) *Habituation*. The test mouse was first placed in the middle chamber and allowed to explore for five minutes with free access to all parts of arena. Each of the two sides contained an empty wire cage (70 × 90 × 70 mm and bars spaced 5 mm apart). (B) *Sociability*. After habituation, an unfamiliar mouse (Stranger 1; a naïve C57BL/6 male) was placed in the wire cage (in the left chamber); another wire cage (in the right chamber) was empty, and the test mouse was placed in the center compartment of the social test box and allowed to explore for a 5-min session, with free access into the two side chambers. The amount of time spent in each chamber and the number of entries into each chamber were measured using the digital video system and ANY-maze software.

### Social avoidance test

Social-avoidance behavior toward a novel C57BL/6 mouse was measured in a two-stage social-interaction test (Chaudhury et al., 2013). In the first 10-min test (target absent), the experimental mouse was allowed to freely explore a square-shaped arena (600 × 600 mm) containing a wire mesh cage (70 × 90 × 70 mm and bars spaced 5 mm apart) placed in the center of the arena. In the second 20-min test, the experimental mouse was reintroduced back into the arena with an unfamiliar C57BL/6 male mouse in a wire mesh cage. Video tracking software (ANY-maze) was used to measure the amount of time the experimental mouse spent in the “interaction zone” (300 × 300 mm). Behavior was measured in mice at 20 min after an intraperitoneal injection of 0.3 ml of oxytocin (OT) (100 ng/kg body weight) or PBS or without any treatment.

### Light-dark transition test

The light-dark transition was measured using the light-dark test chamber, as described by Crawley (2008). The chamber consisted of two dividing rooms. One chamber was brightly illuminated (250 lux), whereas the other was dark (2 lux). Mice were placed into the light arena and were allowed to move freely between the two chambers for 600 s. The light-dark transition test might be useful to predict anxiolytic-like or anxiogenic-like activity in mice. It has the advantages of being quick and easy to use, without requiring the prior training of animals. The test box (200 × 600 × 200 mm) consists of a small dark safe compartment (one-third, 200 × 200 × 200 mm—*dark box*) and a large illuminated aversive compartment (two-thirds, 400 × 200 × 200 mm—*light box*). Each male mouse was placed in the center of the light chamber, and the mouse was allowed to run freely between the two chambers for 10 min. To ensure, the reverse paradigm was also performed: a mouse was placed in the dark chamber first. The trial was recorded for 10 min using the ANY-maze video system. Latency to enter (defined by all four paws entering), time spent, entries and distance traveled in the light chamber were recorded.

### Elevated plus maze

The elevated plus maze is widely used in the study of anxiety-like behavior (Lister, 1987). The maze apparatus was elevated to 50 cm above the floor and consisted of the central platform (5 × 5 cm), from which two open arms (5 × 25 cm) and two closed arms (5 × 25 cm with 15-cm high transparent walls) extended in opposite directions. At least 30 min before each behavioral test, the mice were transported to the testing room for habituation. At the initiation of each session, the mice were placed on the central platform facing an open arm and were allowed to explore the maze freely for 5 min.

### Tail suspension test

The test was performed according to the method previously described (Steru et al., 1985). The mice were individually suspended by the tail above the floor and affixed with adhesive tape placed approximately 1–2 cm from the tip of the tail. The duration of immobility was measured for 6 min. The duration of immobility was defined as the time when mice were completely motionless and hung passively.

### Forced swimming test

The test was performed according to the method described (Porsolt et al., 1977). Mice were placed individually in a cylinder (height 25 cm, diameter 15 cm) filled to a 10-cm depth with water (25 ± 1°C) for 6 min. After the initial 2 min of vigorous activity, the total duration of immobility was recorded during the last 4 min of the test. The duration of immobility was defined as the time during which the mouse remained floating passively, made no attempts to escape and showed only slow movements to keep its head above the water.

The duration of climbing behavior was defined as the time during which the mouse was making forceful thrash movements with its forelimbs against the walls of cylinder during the 6 min.

### Drug treatment

Diazepam was purchased from Wako Pure Chemical Industries, Ltd. (Osaka, Japan), and mirtazapine was obtained from Meiji Seika Pharma Co. Inc. (Tokyo, Japan). Both drugs were dissolved in distilled water with 0.2% of Tween 80. Diazepam was administered at a dose of 1 mg/kg (i.p., 5 ml/kg of body mass). For mirtazapine treatment, the mice were treated once daily for 7 consecutive days at a dose of 1 mg/kg (i.p., 5 ml/kg of body mass). The mirtazapine behavioral test was performed on the eighth day.

### Acoustic startle response and pre-pulse inhibition

A startle reflex measurement system was used (O'Hara & Co., LTD.) to measure startle response and prepulse inhibition. A test session was initiated after placing a mouse in a plastic cylinder, where it was left undisturbed for 10 min. White noise (120 db, 40 ms) was used as the startle stimulus for all trials. The startle response was recorded for 140 ms starting with the onset of the prepulse stimulus. The background noise level in each chamber was 70 db. In the startle and prepulse inhibition paradigm, the prepulse sound was presented 100 ms before the startle stimulus. The intensities of the prepulse sounds were 0, 75, 80, 85, and 90 db, and each sound was separately paired with a 120-db startle sound. The responses in a pair of 0-db prepulse and 120-db startle sound were employed as an acoustic startle response.

### Fear conditioning

Contextual and cued fear conditionings were measured using equipment from O'Hara & Co., LTD. Briefly, training and tests consisted of three trials. During training, the mouse was placed in a test chamber for 300 s. A 60-db white noise (conditioned stimulus) was presented for 30-s, followed by a mild (2 s, 0.3 mA) electric footshock, which served as the unconditioned stimulus. Two more tone-shock stimulus pairings with the same duration as the first stimulus were presented. Context testing was conducted 24 h after conditioning in the same chamber for 300 s. Cued testing with an altered context was conducted 24 h after the contextual test using a different texture and a differently illuminated box. The cued tone (30 s) was presented twice with a 300-s test duration. The animal movement was recorded using a video camera on the ceiling, and the duration of freezing was analyzed using an Image J-based original program.

### Pharmacological models of parkinson's diease

A mouse model of PD was created through intraperitoneal injections of 1-methyl-4-phenyl-1,2,3,6-tetrahydropyridine (MPTP) (Sigma, St Louis, MO, USA; 20 mg/kg) 4 times at 2-h interval (Kühn et al., 2003). Immunohistochemical and biological analysis was performed 4 days after MPTP injection. Briefly, the brain was cut into 10-μ m-thick sections using a cryostat. The sections were blocked in PBS containing 0.3% TritonX-100 and 3% BSA for 1 h at room temperature and incubated at 4°C overnight with primary anti-tyrosine hydroxylase (TH) antibody (Sigma; 1:1000) and anti-GFAP antibody (Millipore, Billerica, MA, USA; 1:500). The sections were subsequently washed with 0.3% TritonX-100 in PBS and incubated with Alexa Fluor 488 (Invitrogen Molecular Probes; 1:200) and Cy3-labled-IgG (Jackson ImmunoResearch Laboratories, West Grove, PA, USA; 1:100) for 1 h at room temperature. Imaging was performed with a Nikon EZ-C1 laser confocal microscope (Tokyo, Japan). TH^+^ neuronal cells in the substantia nigra pars compacta (SNpc) were counted in 2 representative sections (Bregma −3.16 and −3.64 mm) as previously described (Takano et al., 2007). The statistical analysis was performed using Student's *t*-test and One-way or Two-Way ANOVA followed by Bonferroni/Dunn test.

For western blot analysis, brain samples from the striatum were solubilized in buffer containing 1% NP40, 0.1% SDS and 0.2% deoxycholate and were subjected to western blotting with the following antibodies: TH (Sigma), α-synuclein (Santa Cruz), GFAP (Millipore) and β-actin (Sigma). Primary antibody binding was visualized using the ECL system (GE Healthcare Bio-Sciences Corp., Piscataway, NJ, USA). Quantification of each band was performed using Image J (version 1.42, Wayne Rasband, National Institutes of Health, MD, USA).

### Blood and tissue collection

Male mice were anaesthetized by intraperitoneal injection of pentobarbitone (35 mg/Kg). Blood samples of 1 ml were collected by cardiac puncture and centrifuged at 1600× g for 15 min at 4°C. Plasma samples (~200–400 μl/mouse) were collected and stored at −80°C until use.

The whole pituitary and hypothalamus were removed, and homogenized in 10 mM Tris-base (pH 7.4) using a 1-ml Teflon/glass homogenizer. The fresh homogenates were used for determining ADP-ribosyl cyclase activity, as described (Jin et al., 2007).

The whole pituitary and hypothalamus were removed and homogenized in 0.4 M acetic acid, using a 1-ml Teflon/glass homogenizer. The fresh homogenates were centrifuged at 800× g for 20 min at 4°C. The supernatants were collected and stored at −80°C until measurement of oxytocin. Protein content was determined using a Bio-Rad protein assay kit and bovine serum albumin as a standard (Bio-Rad, Hercules, CA, USA).

### Enzyme immunoassay for oxytocin

To determine the concentrations of OT in the plasma and brain tissues, an OT immunoassay kit was used according to the manufacturer's protocol (Assay Designs Inc., Ann Arbor, MI, USA), as described previously (Jin et al., 2007).

### ADP-ribosyl cyclase assay

Assay to detect ADP-ribosyl cyclase activity in the hypothalamus and pituitary was performed in whole homogenates using the nicotinamide guanine dinucleotide technique as described previously (Jin et al., 2007; Graeff and Lee, 2013). Briefly, 2 ml of reaction mixtures containing 60 μ M NGD^+^, 50 mM Tris-HCl, pH 6.6, 100 mM KCl, 10 μ M CaCl_2_, were maintained at 37°C with constant stirring. The samples were then excited at 300 nm, and fluorescence emission was continuously monitored at 410 nm in a Hitachi 650 spectrofluorometer. Activity was calculated from the linear portion of the 10 min time course by fitting a linear function to the data points recorded every 15 s.

The specific ADP-ribosyl cyclase activity was calculated using cyclic GDP-ribose (cGDPR) standard, and the results are presented as nanomoles cGDPR per min per mg of protein.

### RT-PCR

Total RNA was isolated from mouse spleens and brain subregions using TRIzol Reagent (Invitrogen, Carlsbad, CA, USA). cDNA was synthesized from 0.5 μ g of total RNA using the ReverTra Ace-α (Toyobo, Osaka, Japan) according to the manufacturer's protocol. PCR was performed on a Mastercycler ep gradient S (Eppendorf, Hamburg, Germany) using the following conditions: 1 cycle of 94°C for 30 s, followed by 25 or 30 cycles of 94°C for 30 s, 58°C for 30 s and 72°C for 40 s, with a final extension step at 72°C for 1 min. The primer sequences used have been reported (Itoh et al., 1998). RT-PCR products were separated electrophoretically on 1.2% gels and stained with ethidium bromide. Band intensity was measured from photographs using Image J. The data are shown as *CD157 i*ntensity divided by the β-actin intensity of the same sample.

### Immunohistochemistry

Prior to immunohistochemical procedures, the brain sections were washed in PBS and were incubated in PBS containing 0.3% Triton X-100 (Sigma) and 10% normal horse serum (Invitrogen) for 1 h. Subsequently, sections were incubated with anti-NeuN (1:200; Chemicon, Temecula, CA, USA) antibody at room temperature for 12 h. The samples were washed with PBS and incubated with the Alexa Fluor 488 dye (1:200) at room temperature for 3 h. After washing with PBS, the sections were incubated with 10 mg/ml Hoechst 33258 solution (Sigma) for visualization of the nucleus. Imaging was performed using an Axio Observer.A1 (Zeiss, Jena, Germany).

The amydgala sections were first incubated with primary anti-murine CD157 antiserum and subsequently washed with 0.3% TritonX-100 in PBS and incubated with goat anti-rabbit IgG Alexa Fluor 488 (1:200) and DAPI (Dojindo; 1:2000) for 1 h at room temperature. Subsequently, the sections were washed with PBS containing 0.3% TritonX-100 and coverslipped with anti-fade (Akimoto et al., 2013). Imaging was performed using an Olympus IX71 microscope. Images were analyzed with MetaMorph software (Molecular Devices, Downingtown, PA, USA) (Higashida et al., 2004).

### Nissl staining

Frozen brain sections were stained with 0.5% thionin acetate (Nacalai Tesque) at room temperature for 1 min. Low-magnification images were taken with the BZ-9000 Generation II microscope (Keyence, Osaka, Japan).

### c-FOS immunoreactivity

After the 10-min open field test, the mice were sacrificed immediately for brain fixation with 4% paraformaldehyde in PBS overnight. After the brains were cryoprotected in PBS containing 15 and 30% sucrose, the brains were cut into 20-μ m-thick sections using a freezing microtome. The sections were processed as described above and incubated overnight at 4°C with the primary antibody c-Fos (Santa Cruz, Dallas, TX, USA; 1:200). The brain sections were stained with goat anti-rabbit IgG Alexa Fluor 488 (1:200) and DAPI (1:2000) for 1 h at room temperature. Subsequently, the sections were washed with PBS containing 0.3% TritonX-100 and coverslipped with anti-fade. Imaging was performed using an Olympus IX71 microscope. Images were recorded with MetaMorph software. c-Fos-positive cells were counted manually using the following parameters: fluorescence diameters <13.5 μm and intensities >498.6 were counted. The average intensity of fluorescence was within a 5-fold range.

### Statistical analysis

The data are expressed as the means ± s.e.m. The comparisons were evaluated between two groups (*CD157*^+/+^ or *CD157*^−/−^) using Student's *t*-test or One-Way ANOVA, followed by *post-hoc* Bonferroni test. In all analyses, *P* < 0.05 indicated statistical significance.

## Results

### General health and locomotor activities

We used *CD157*^−/−^ mice newly reproduced from frozen *CD157*^+/−^ eggs (Itoh et al., 1998). These mice expressed no CD157 immunoreactivity in the white pulp of the spleen (data not shown). *CD157*^−/−^ mice developed normally compared with wild-type (*CD157*^+/+^) C57BL/6 mice and were viable, as previously described (Itoh et al., 1998), although the weaning rate of 3- to 4-week-old pups was low (approximately 50% of wild-type mice). We did not detect skin lesions due to self-injurious over-grooming behavior in the *CD157*^−/−^ mice, suggesting no repetitive behavior.

**Figure 1:**
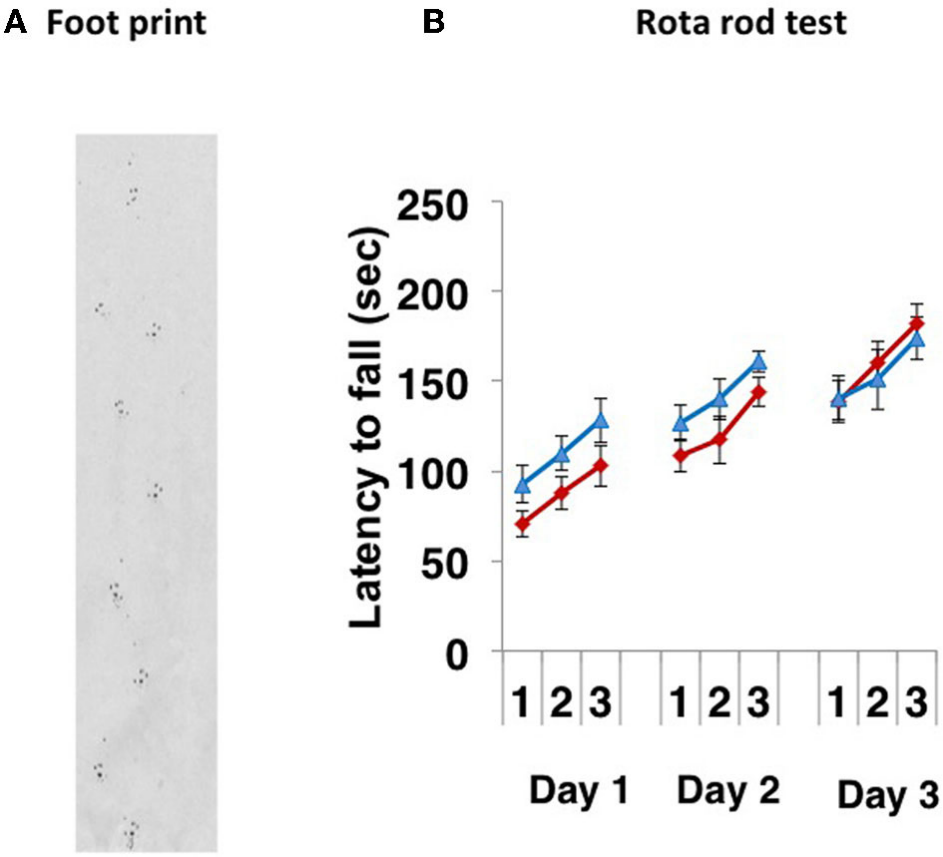
**Motor coordination tests**. **(A)** A representative footprint. The footprint patterns revealed no impairment of motor coordination in *CD157*^−/−^ mice (*n* = 20). **(B)** Motor learning and memory was increased following training (3 trials a day for 3 days) similarly in both types of *CD157*^+/+^ (blue triangles, *n* = 10) and *CD157*^−/−^ (red diamonds, *n* = 20) mice in the accelerating (5–40 rpm) rota rod paradigm.

Paralysis, tremor, rigity, postual abnormality and involuntary movements were not observed. The * CD157^−/−^* mice walked normally (Figure 1A). When 8- to 10-week-old *CD157*^+/+^ and *CD157*^−/−^ male mice were subjected to the rota rod test for 3 successive days, there were no differences in their motor coordination and learning (Figure 1B). The latency to fall from the rotating rod was similar between the two genotypes, suggesting that there was no weakness in the fore and hind limbs and no coordinate movement dysfunction.

**Figure 2:**
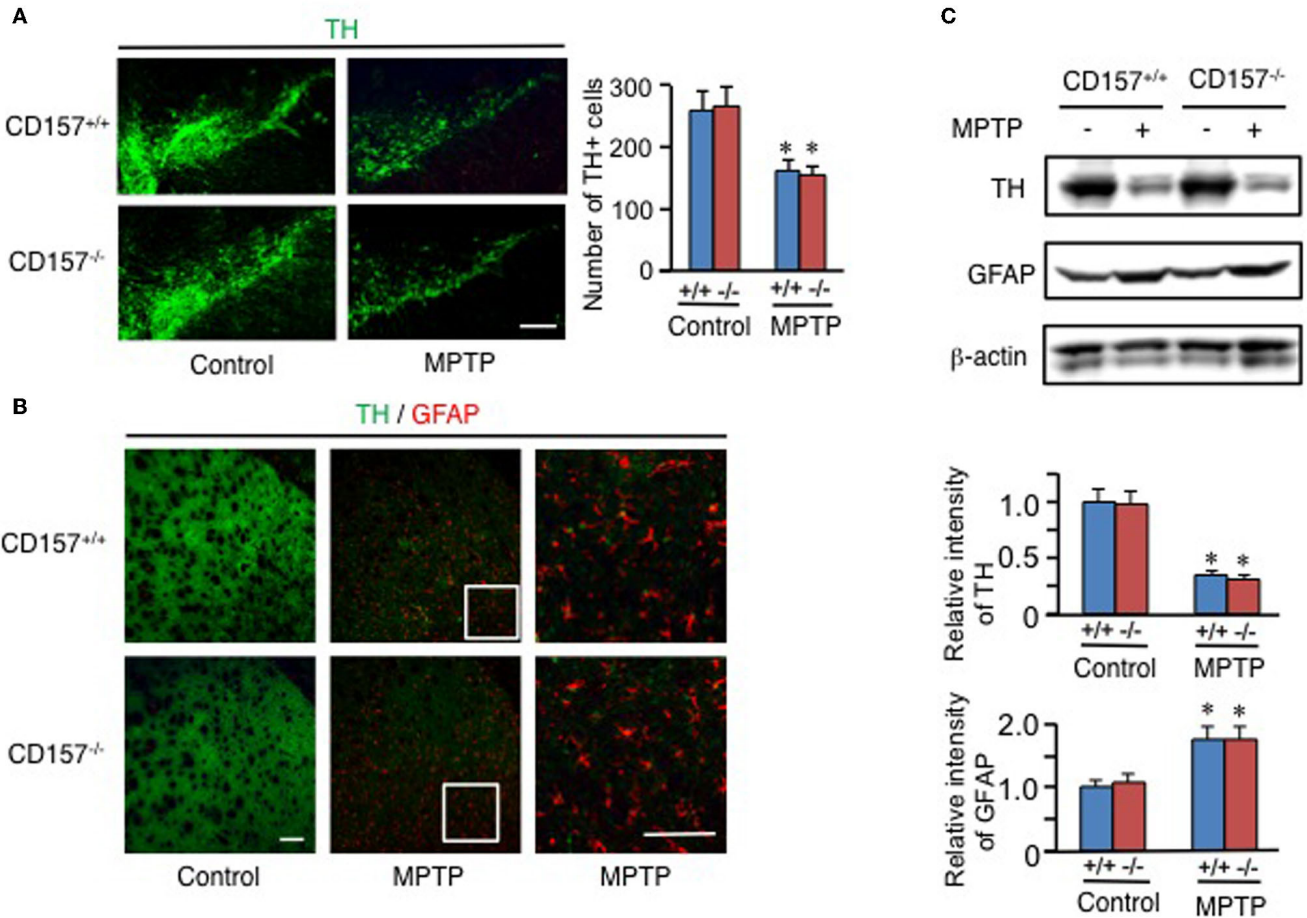
**Degeneration of nigro-striatal neurons after MPTP administration. (A,B)** Brain sections, including the substantia nigra **(A)** or striata **(B)** from *CD157*^+/+^ (+/+) or *CD157*^−/−^ (−/−) mice injected with MPTP or vehicle, were immunostained with anti-tyrosine hydroxylase (TH) and anti-GFAP antibodies. The numbers of TH^+^ cells in the substantia nigra pars compacta were counted and are shown in the graph **(A)**. The values shown represent the means ± s.e.m. of four experiments. ^*^P < 0.05 compared with vehicle-administered mice. Scale bar = 50 μm. **(C)** Protein extracts from the striata of *CD157*^+/+^ or *CD157*^−/−^ mice that were injected with MPTP or vehicle were subjected to western blotting with the indicated antibodies. The relative intensities are shown in the graphs. The intensities of signals derived from mice with vehicle administration were designated as one. The values shown represent the means ± s.e.m. of four experiments. ^*^P < 0.05 compared with vehicle-administered mice.

**Figure 3:**
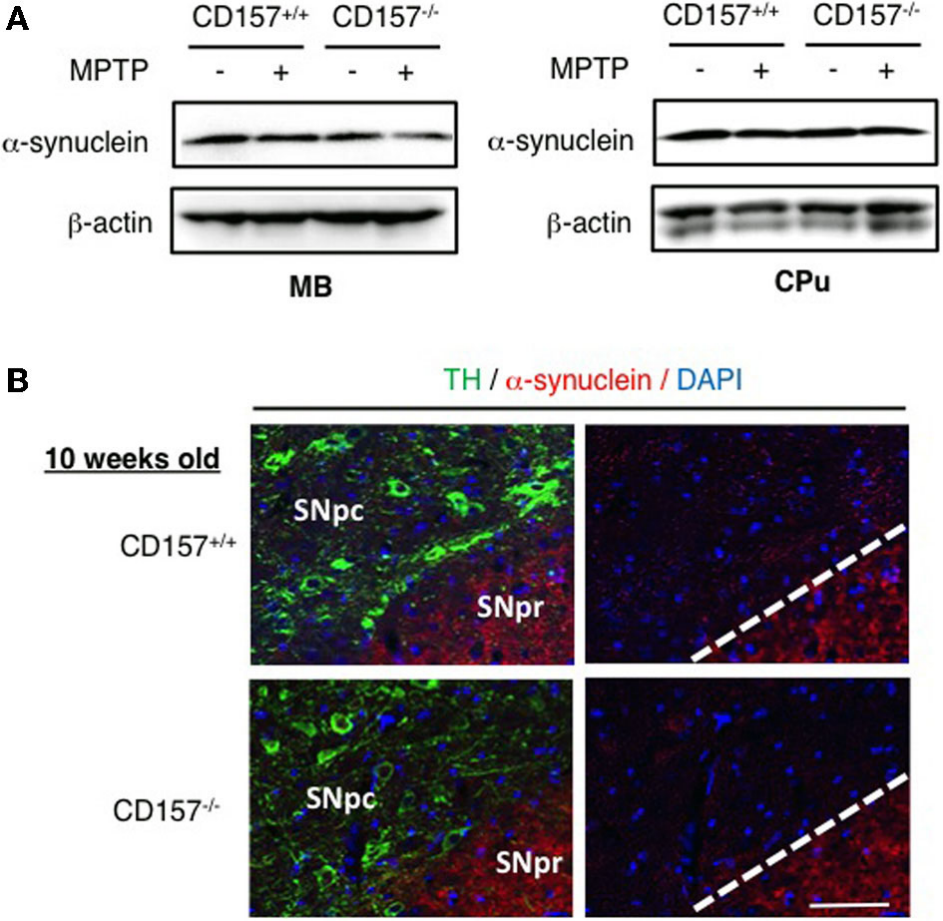
**Expression of α-synuclein. (A)** Protein extracts from the ventral midbrain or caudate putamen of *CD157*^+/+^ or *CD157*^−/−^ mice that were injected with MPTP or vehicle were subjected to western blotting with anti-α-synuclein antibody and anti-β-actin-antibody. **(B)** Brain sections including the substantia nigra pars compacta (SNpc) and reticulate (SNPr) from *CD157*^+/+^ or *CD157*^−/−^ mice that were injected with MPTP or vehicle were immunostained with anti-TH antibody and anti-α-synuclein antibody. 10 week-old mice. Scale bar = 50 μm.

One of the hallmarks in PD is the degeneration of dopaminergic neurons in the substantia nigra. Therefore, we conducted an analysis of tyrosine hydroxylase-positive cells by immunohistochemistry or by analysing the mRNA expression level of tyrosine hydroxylase in both genotypes (Figure 2). Essentially no difference was found in wild-type and knockout mice. In addition, when the acutely 1-methyl-4-phenyl-1,2,3,6 tetrahydropyridine (MPTP)-treated loss of tyrosine hydroxylase-positive cells in the substantia nigra and the striata was examined. MPTP-treated *CD157*^−/−^ mice exhibited the same sensitivity as wild-type mice with regard to nigro-striatal degeneration in the MPTP-induced PD model. Northern blotting showed that the mRNA expression level of α-synuclein did not differ between the two MPTP-treated genotypes (Figure 3). Therefore, the results show that *CD157*^−/−^ mice have little to no deficiency in motor learning, memory and function in young adulthood, though the more chronic mouse PD model or aged mice should be used to unravel any possible long-term effect of altered CD157 function.

**Figure 4:**
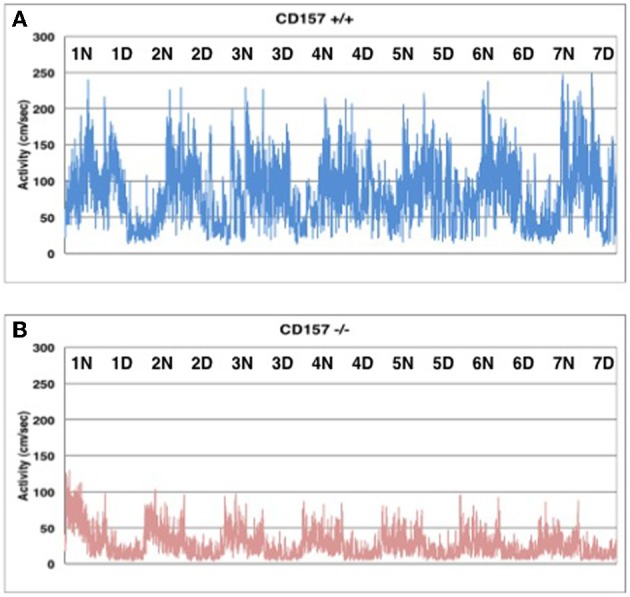
**Spontaneous homecage activity in *CD157*^+/+^ (A) and *CD157*^−/−^ (B) mice**. Activity was monitored in a 24 h record for 7 days (*n* = 8, each genotype). Higher activity levels indicate the dark phase during a 12:12 light:dark cycle (lights on at 8:45 AM; lights off at 8:45 PM).

Interestingly enough, home cage activity monitoring revealed that the activity level of the *CD157*^−/−^ mice during both the day and night was approximately 40 ± 4% of that of the *CD157*^+/+^ mice (*n* = 8 in each genotype, *t* = 7.575, *P* < 0.05; Figure 4). The day and night rhythms were similar between wild-type and knockout mice during the time period of 7 days, Therefore, we estimated that the *CD157*^−/−^ mice may squat in one place for long periods of time, which may lead to decreases in total motor activities in daily life. Because this characteristic behavior seems to be derived from specific emotional conditions, we assessed their psychological responses in a series of emotion-related social behavior tests.

### Behaviors during exposure to novelty

**Figure 5:**
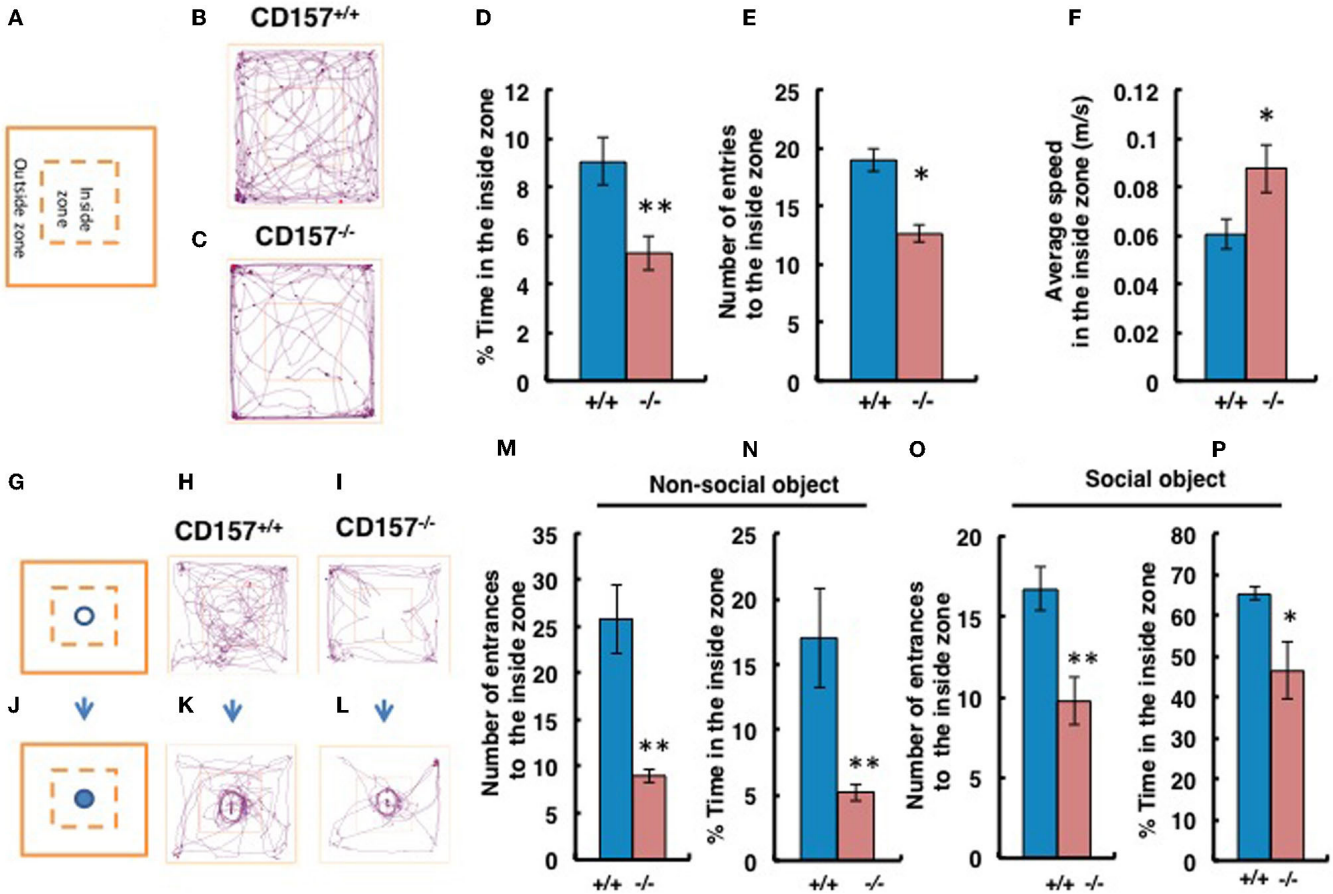
**Anxiety and social preference tests in the open field with adult male mice**. Schemes of experiments illustrate the open field without any object **(A)** or with a non-social target **(G)** or a social (male mouse) target **(J)** in the center in the inside zone. Representative movement tracks of the *CD157*^+/+^ and *CD157*^−/−^ mice in the open field with no object **(B,C)**, with the non-social target **(H,I)** or with the social target **(K,L)** over a 10-min period are shown. Percentage of time spent in the inside zone **(D)**, number of entries to the inside zone **(E)** and average speed in the inside zone **(F)** with no object are shown. Number of entries to the inside zone **(M,O)** and percentage of time spent in the inside zone **(N,P)** with the non-social or social object are shown. The blue bars represent the *CD157*^+/+^ mice, and the red bars represent the *CD157*^−/−^ mice. The data are expressed as the means ± s.e.m., *n* = 8, ^*^P < 0.05 or ^**^P < 0.01.

When mice were exposed to the novel environment in the open field apparatus (Figure 5A), judging from the observed tracks traveled, *CD157*^−/−^ male mice engaged less in exploratory behavior, particularly in the inside zone, than wild-type males (Figures 5B,C). Although there was little to no behavioral difference observed in the outside zone (data not shown), the percentage of time in the inside zone was reduced for *CD157*^−/−^ mice (*n* = 8, *t* = 3.144, *P* < 0.01; Figure 5D). *CD157*^−/−^ mice entered the inside zone fewer times than did *CD157*^+/+^ mice (*n* = 8, *t* = 2.613, *P* < 0.05; Figure 5E). In contrast, the average speed of the *CD157*^−/−^ mice in the inside arena was higher than that of the wild-type mice (*n* = 8, *t* = 2.127, ^*^P < 0.05; Figure 5F).

We performed social interaction and preference tests with novel non-social or social targets in the same open field apparatus. First, using a non-social object (Figures 5G–I), we confirmed that the *CD157*^−/−^ male mice exhibited a higher level of anxiety due to their reduced entry into the inside zone (*n* = 8, *t* = 4.593, *P* < 0.01; Figure 5M). The percentage of time spent close to the non-social object in the inside zone was significantly shorter for *CD157*^−/−^ mice than for *CD157*^+/+^ mice (*n* = 8, *t* = 3.822, *P* < 0.01; Figure 5N). When an unknown male mouse was used as a social stimulus (Figures 5J–L), the knockout mice showed increased interest compared with their interest in the non-social object (Time in the inside zone in Figure 5N vs. Figure 5P, *t* = 4.548, *P* < 0.001). However, the *CD157*^−/−^ mice resulted in less entry into the inside zone (*n* = 8, *t* = 2.333, *P* < 0.01; Figure 5O) and a shorter time spent in the inside zone (*n* = 8, *t* = 3.135, *P* < 0.05; Figure 5P) compared with the wild-type mice. The results to the novelty indicate that the *CD157*^−/−^ mice have a higher level of anxiety in the novel environment, as demonstrated by a preference to be near a protective wall rather than exposed to danger in the open field and lower sociability to the novel non-social and social targets.

**Figure 6:**
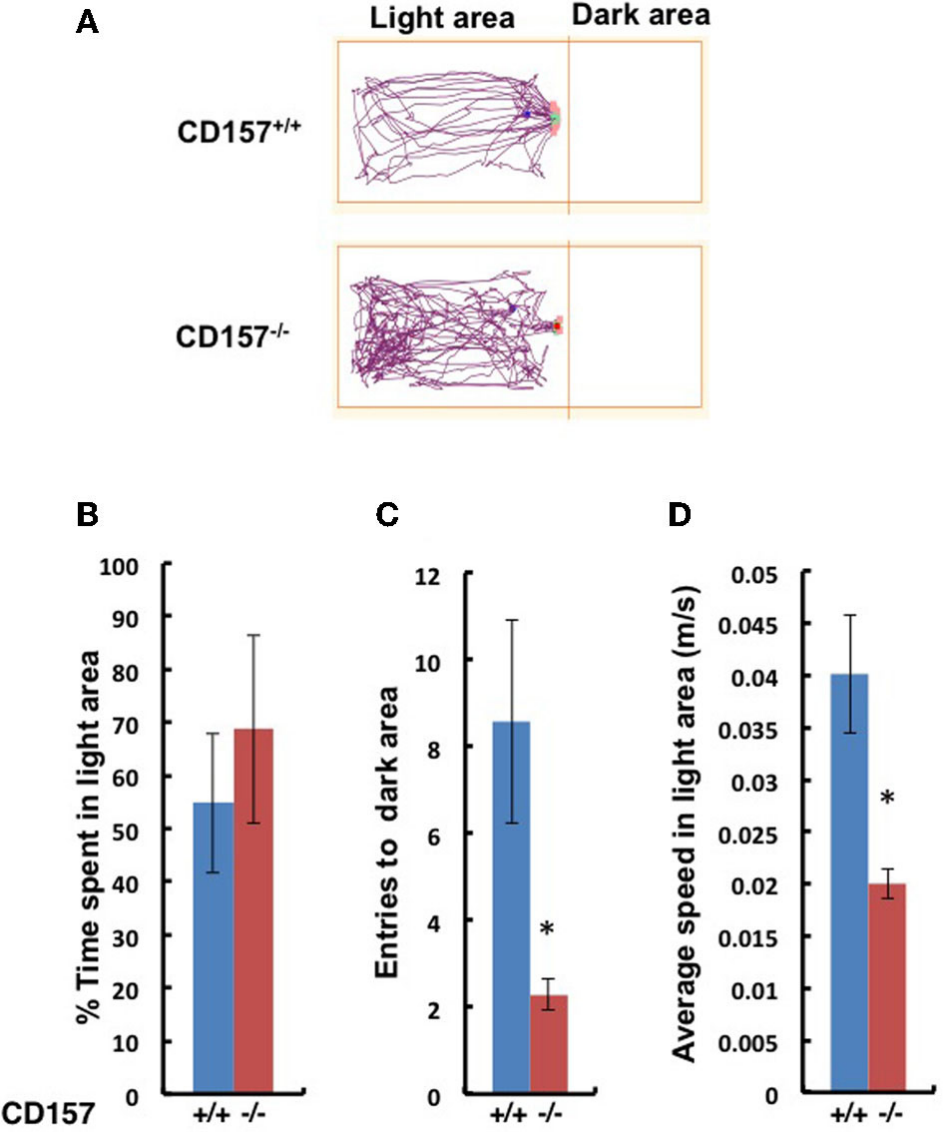
**Behavior of wild-type (blue, +/+) or CD157 knockout (red, −/−) mice in the light-dark transition test. (A)** Representative traces of both genotypes in the light compartment for the first 5 min. The percentage of time spent in the light compartment **(B)**, transition numbers between the two compartments **(C)** and the average speed in the light compartment **(D)**. The results are expressed as the means ± s.e.m. ^*^P < 0.05 compared with *CD157*^+/+^ group, *n* = 8.

This anxiety-related behavior was confirmed using the light and dark chamber test (Figure 6). The transition from the light to dark arena was significantly different (Figures 6A,B). The *CD157*^−/−^ mice entered the dark chamber with an average frequency of 2.2 ± 0.4 times during a 10-min test, while the *CD157*^+/+^mice entered at a frequency of 8.6 ± 2.4 times during the same time span (*n* = 8, *P* < 0.01; Figure 6C). While in the light zone, the *CD157*^−/−^ mice moved significantly more slowly than the *CD157*^+/+^ mice (*n* = 8, *P* < 0.05; Figure 6D). Reversely, when the mice were first placed in the dark arena prior to initiating the experiments, the * CD157^−/−^* mice stayed in the dark for a longer period of time (*n* = 10, data not shown). These results indicate that the *CD157*^−/−^ mice experience anxiety when transitioning into a novel condition.

### Anxiety-related behaviors and recovery

**Figure 7:**
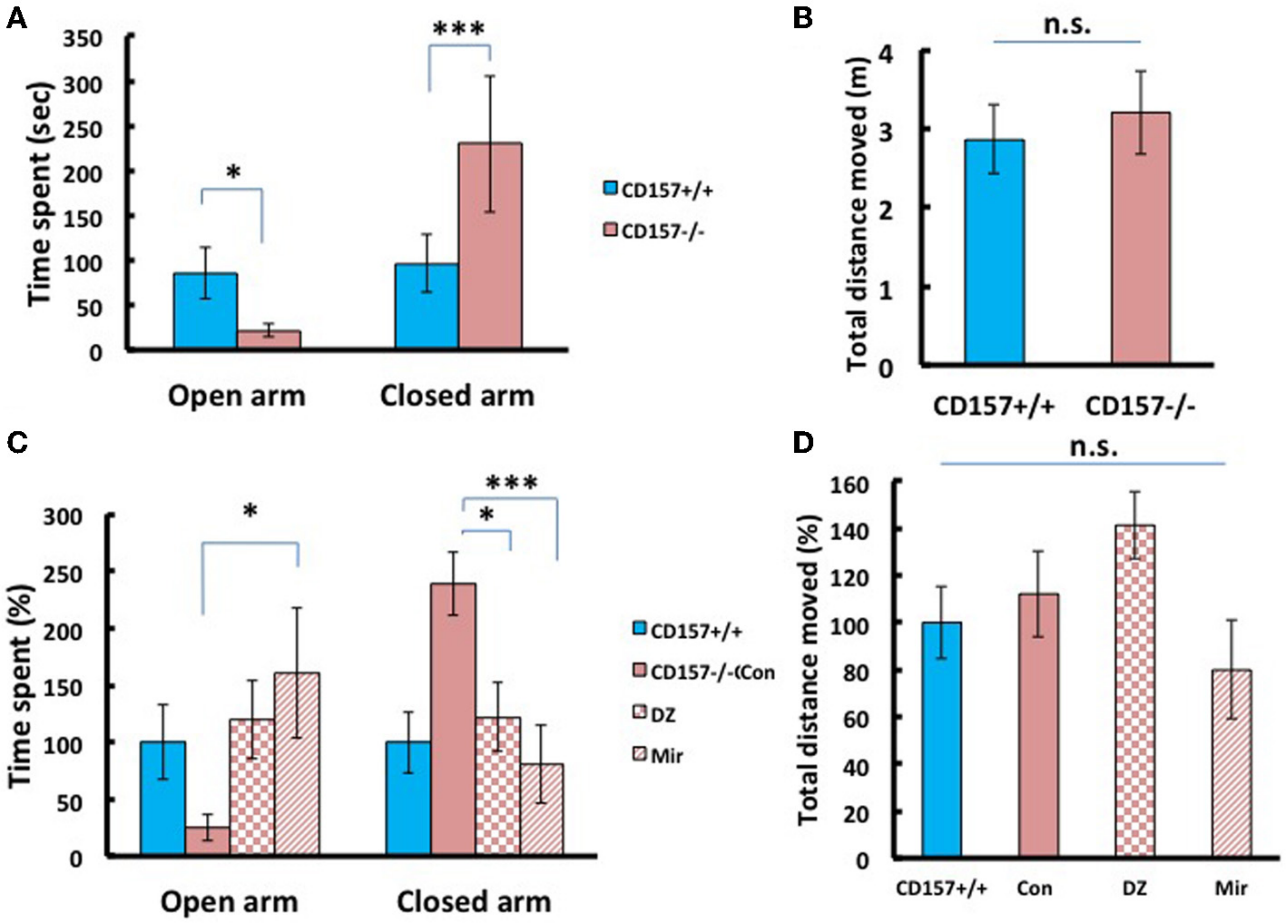
**Anxiety-related behavior of adult male mice in the elevated plus maze**. **(A)** *CD157^−/−^* mice spent a significantly shorter time in open arms (^*^P < 0.05) and a longer time in closed arms (^***^P < 0.005) compared with *CD157*^+/+^ mice (*n* = 9–10 per group, *t*-test). **(B)** The total distance moved in the elevated plus maze was not different between the two groups. **(C)** Effects of diazepam (DZ, 1 mg/kg, i.p.) and mirtazapine (Mir, 1 mg/kg, i.p.) on *CD157*^−/−^ mice. Treatment with mirtazapine for 7 days significantly increased the time spent in open arms in *CD157*^−/−^ mice (*n* = 5–10 per group, One-Way ANOVA followed by Bonferroni's *post-hoc* test, ^*^P < 0.05). Diazepam and mirtazapine significantly decreased the time spent in closed arms for *CD157*^−/−^ mice (One-Way ANOVA followed by Bonferroni's *post-hoc* test, ^*^P < 0.05, ^***^P < 0.005, respectively). **(D)** The total distances moved were not different between the three groups. The data are expressed as the means ± s.e.m. The values are designated as 100% in non-treated wild-type mice.

Next we applied the elevated plus maze test, the most standardized experiment for measuring anxiety in a mouse model. As shown in Figure 7, *CD157*^−/−^ mice stayed only briefly in the open arm (Figure 7A; *P* < 0.05) and longer in the closed arm (*P* < 0.005), compared with the wild-type mice, with no differences in total distance (Figure 7B), indicating anxiety-related behaviors in the knockout mice.

To determine whether these anxiety-related behaviors observed in *CD157*^−/−^ mice can be rescued through pharmacological treatment, we examined diazepam (Altamura et al., 2013), an anti-anxiety drug that activates GABA_A_ receptors and mirtazapine (Watanabe et al., 2011), a second-generation anti-depressant with a combined serotonergic and noradrenergic mechanism. Based upon preparatory experiments for determining drug doses, we used diazepam with a concentration of 1 mg/kg in a single i.p. administration prior to 30 min of testing, or mirtazapine at 1 mg/kg, i.p., once a day for 7 consecutive days.

For time spent in open arms, One-Way ANOVA followed by Bonferroni's *post-hoc* test revealed a significant effect of the drugs in *CD157*^−/−^ mice [mirtazapine (*n* = 10) and diazepam (*n* = 5); *F*_(2, 18)_= 5.251, *P* < 0.05; Figure 7C]. For time spent in closed arms One-Way ANOVA followed by Bonferroni's *post-hoc* test showed the significant effects of these drugs in *CD157*^−/−^ mice [*F*_(2, 18)_= 8.495; *P* < 0.05 or *P* < 0.005, respectively]. Both drugs rescued knockout mice to the behavioral level observed in the untreated mice. The total distances moved in the apparatus were not different in treated and untreated groups (Figure 7D). These experiments indicate the anxiety behaviors are measurable and robust in *CD157*^−/−^ and pharmacologically reversible to the control mouse level.

### Depression-like behavior and recovery

**Figure 8:**
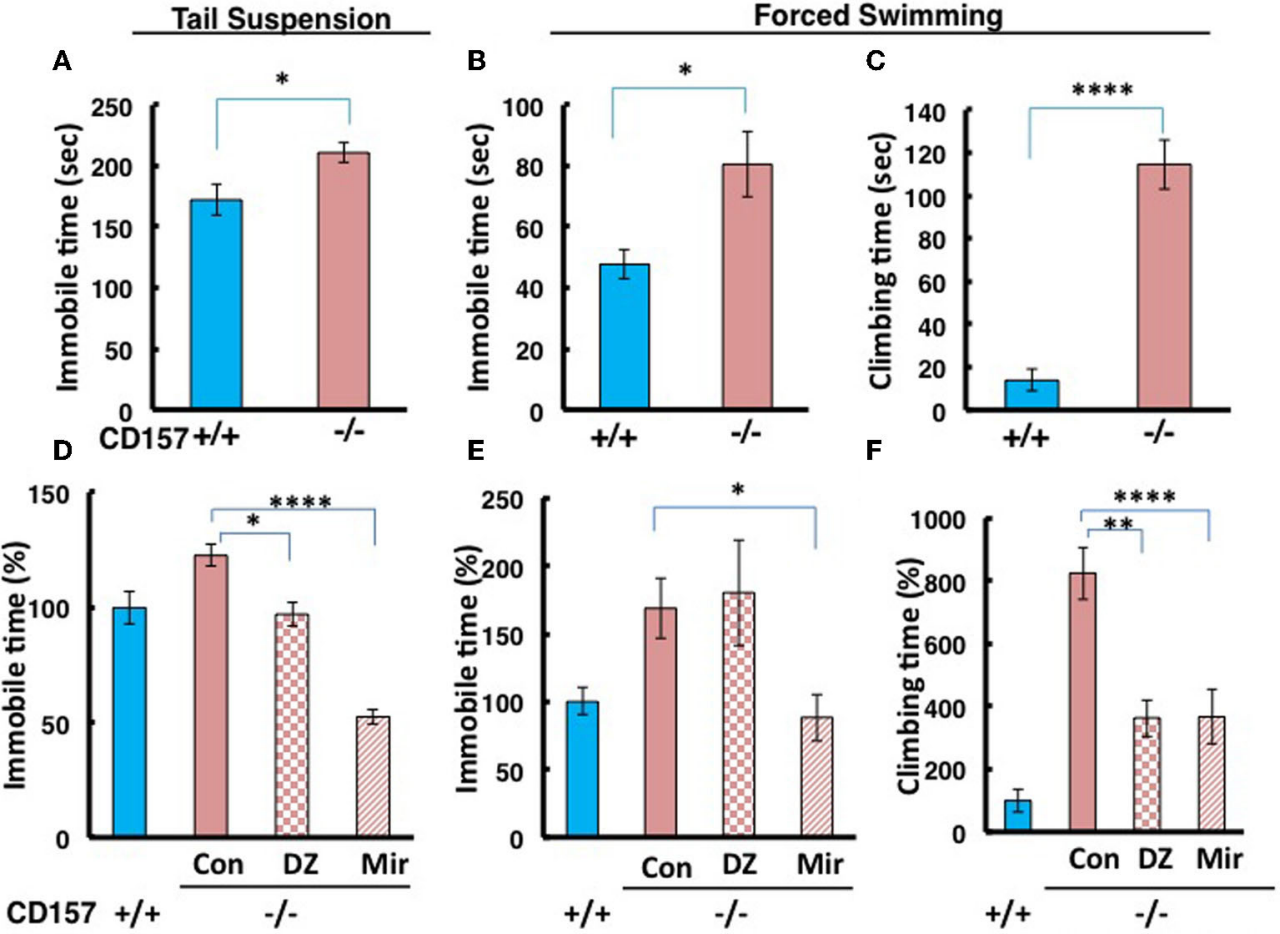
**Depression-like behavior in tail suspension and forced swimming tests. (A)** The time of immobility in the tail suspension test was longer for *CD157*^−/−^ mice compared with *CD157*^+/+^ mice (*n* = 20 per group, ^*^P < 0.05). **(B,C)** The immobility or climbing time in the forced swimming test was longer for *CD157*^−/−^ mice compared with *CD157*^+/+^ mice (*n* = 10 per group, ^*^P < 0.05 or ^****^P < 0.001, respectively). The data are expressed as the means ± s.e.m. **(D–F)** Depression-like behavior was recovered at 30 min after a single i.p. administration of diazepam (DZ, 1 mg/kg) or after chronic administration of mirtazapine for 7 days (Mir, 1 mg/kg, i.p.) in tail suspension **(D)** or forced swimming **(E,F)** tests in *CD157*^−/−^ mice. The data are expressed as the means ± s.e.m. The values are designated as 100% in non-treated WT mice (ANOVA followed by Bonferroni's *post-hoc* test, *n* = 5–10 mice per group; ^*^P < 0.05, ^**^P < 0.01, and ^****^P < 0.001, respectively).

To make the lower movement activity during day and night in knockout mice much clear, we examined depression-like behaviors using tail suspension and forced swimming tests. We observed a significant increase in immobility during the tail suspension and forced swimming tests in *CD157*^−/−^ adult male mice compared with *CD157*^+/+^ mice [tail suspension, *n* = 20, *t* = 2.583, *P* < 0.05 (Figure 8A); forced swimming, *n* = 10, *t* = 1.689, *P* < 0.05 (Figure 8B)].

Interestingly, the *CD157*^−/−^ mice showed a peculiar behavior. They took a significantly longer time to climb up the wall of the swimming pool (*t* = 7.925, *P* < 0.001; Figure 8C). While this climbing behavior was frequently observed during the first 2 min and quickly adapted and stopped struggling in the water in wild-type mice, *CD157*^−/−^ mice continued to attempt to climb the wall from the start to the end of the observation period (6 min) to escape, potentially reflecting an altered emotional state.

In the tail suspension test, One-Way ANOVA followed by Bonferroni's *post-hoc* test revealed significant recovery to the levels of wild-type mice or more in *CD157*^−/−^ mice using the two drugs [*F*_(2, 27)_ = 27.904, *P* < 0.05, *P* < 0.001, respectively; Figure 8D]. In contrast, immobility in the forced swimming test was significantly rescued to the control level after treatment with mirtazapine, but not diazepam, in *CD157*^−/−^ mice [One-Way ANOVA followed by Bonferroni's *post-hoc* test; *F*_(2, 22)_ = 4.627, *P* < 0.05; Figure 8E]. The climbing time in the forced swimming test was recovered from approximately 800–350% of the levels in the untreated wild-type mice using both drugs [One-Way ANOVA followed by Bonferroni's *post-hoc* test, *F*_(2, 22)_ = 10.167, *P* < 0.01 or 0.001, respectively; Figure 8F]. This result shows that depression-like behavior is possible in *CD157*^−/−^ mice, which is reversible by classical and new types of anti-anxiety or anti-depression drugs.

### Sociability in the three-chamber test

**Figure 9:**
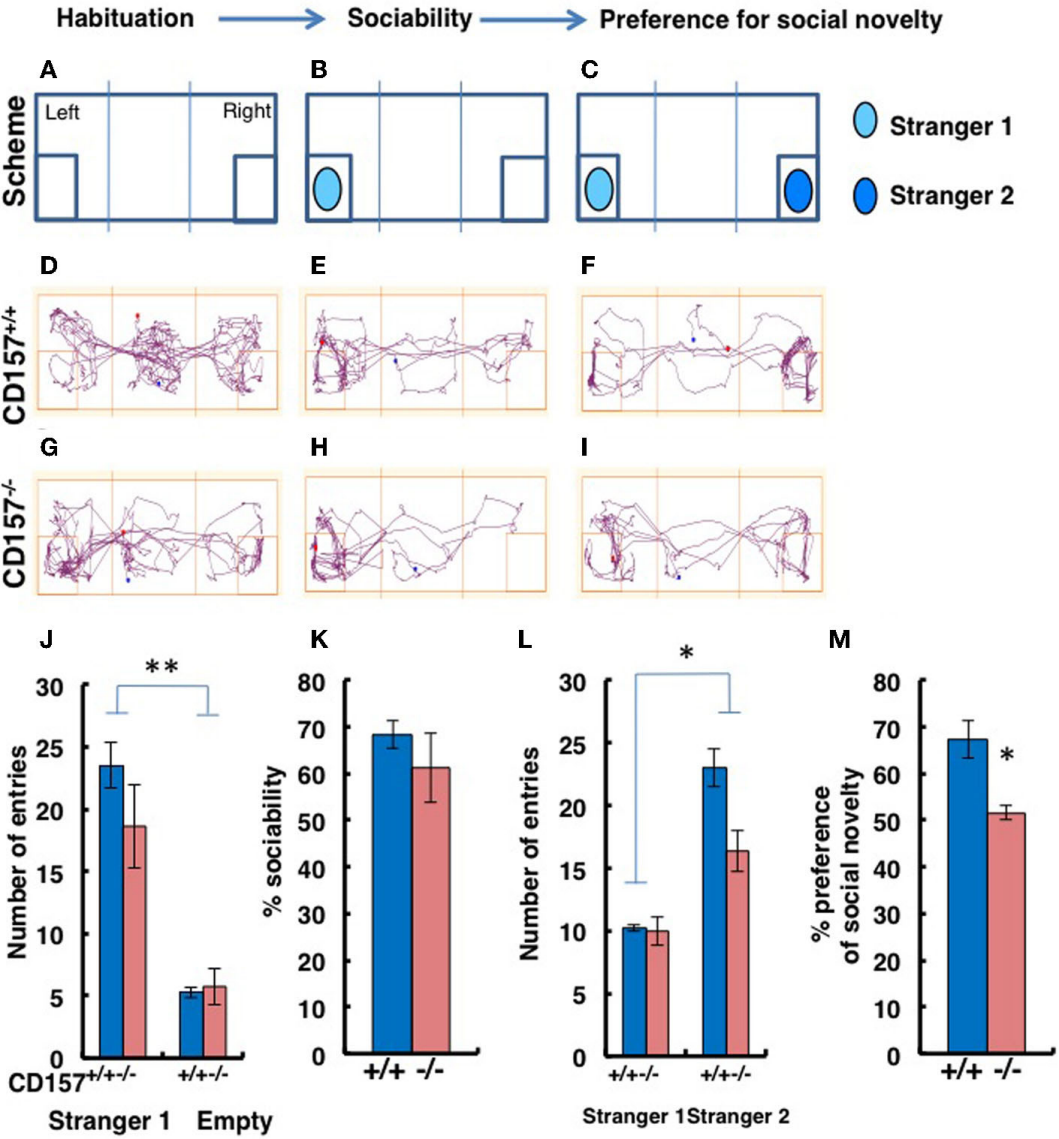
**Sociability and social preferences of adult male mice in the three-chamber apparatus**. **(A–I)** Experimental schemes and representative tracks of the *CD157*^+/+^ and *CD157*^−/−^ mice in the sociability task when a stimulus male mouse was placed in the left chamber **(B,E,H)** or in the social novelty task when a new target male mouse was placed in the right chamber **(C,F,I)**. The number of entries into Stranger 1 and empty chambers **(J)** or the Stranger 1 and Stranger 2 chambers **(L)**, the percentage sociability **(K)** and the social novelty **(M)** of the *CD157*^+/+^ (blue) and *CD157*^−/−^ (red) mice are shown. The data are expressed as the means ± s.e.m., *n* = 8, ^*^P < 0.05, ^**^P < 0.01, respectively.

Although we had already performed tests of sociability using novel targets in the open field test, to confirm the abnormal sociability phenotype in *CD157*^−/−^ mice, we used a more specific tool, a three-chamber box test (Figure 9). For these experiments, a target male mouse was placed in the left chamber (Stranger 1, Figure 9B), and a second target male mouse was placed in the right chamber (Stranger 2, Figure 9C). The mice displayed no significant side-preference during the habituation phase (Figures 9A,D,G). Representative traces are shown for the both types of mice during the sociability session (Figures 9B,E,H) and the preference of social novelty session (Figures 9C,F,I). There was no difference in the number of entries to the Stranger 1 chamber by the *CD157*^−/−^ and *CD157*^+/+^ mice (*n* = 8; Figure 9J). The number of entries to the chamber containing the social target (Stranger 1) was equally and significantly higher than the number of entries to the empty room [Two-Way ANOVA, *F* = 73.673, *P* < 0.01; Figure 9J]. The percentage of sociability, which was estimated from the percentage of time that the mouse stayed in the target room, was also equally high for both genotypes (*n* = 8; Figure 9K).

When the second target mouse was placed in the right chamber, the number of entries to the new social target (Stranger 2) chamber by the *CD157*^−/−^ mice was lower than the number of entries made by the *CD157*^+/+^ mice [*n* = 8; Two-Way ANOVA for effect of genotype, *F* = 11.057, *P* < 0.05; Figure 9L]. In addition, the numbers of entries for both mouse strains were much higher than the numbers of entries to the room containing Stranger 1 (*P* < 0.05; Figure 9L). The percentages of time spent with Stranger 2 were 51.6 ± 1.7% for CD157^−/−^ mice *vs*. 67.1 ± 3.9% for *CD157*^+/+^ mice (*n* = 8, *t* = 3.067, *P* < 0.05; Figure 9M). Therefore, it is reasonable to conclude that the *CD157*^−/−^ mice demonstrated the same level of sociability, with social avoidance and/or decreased social preference.

### Responses to fear and startle stimuli

**Figure 10:**
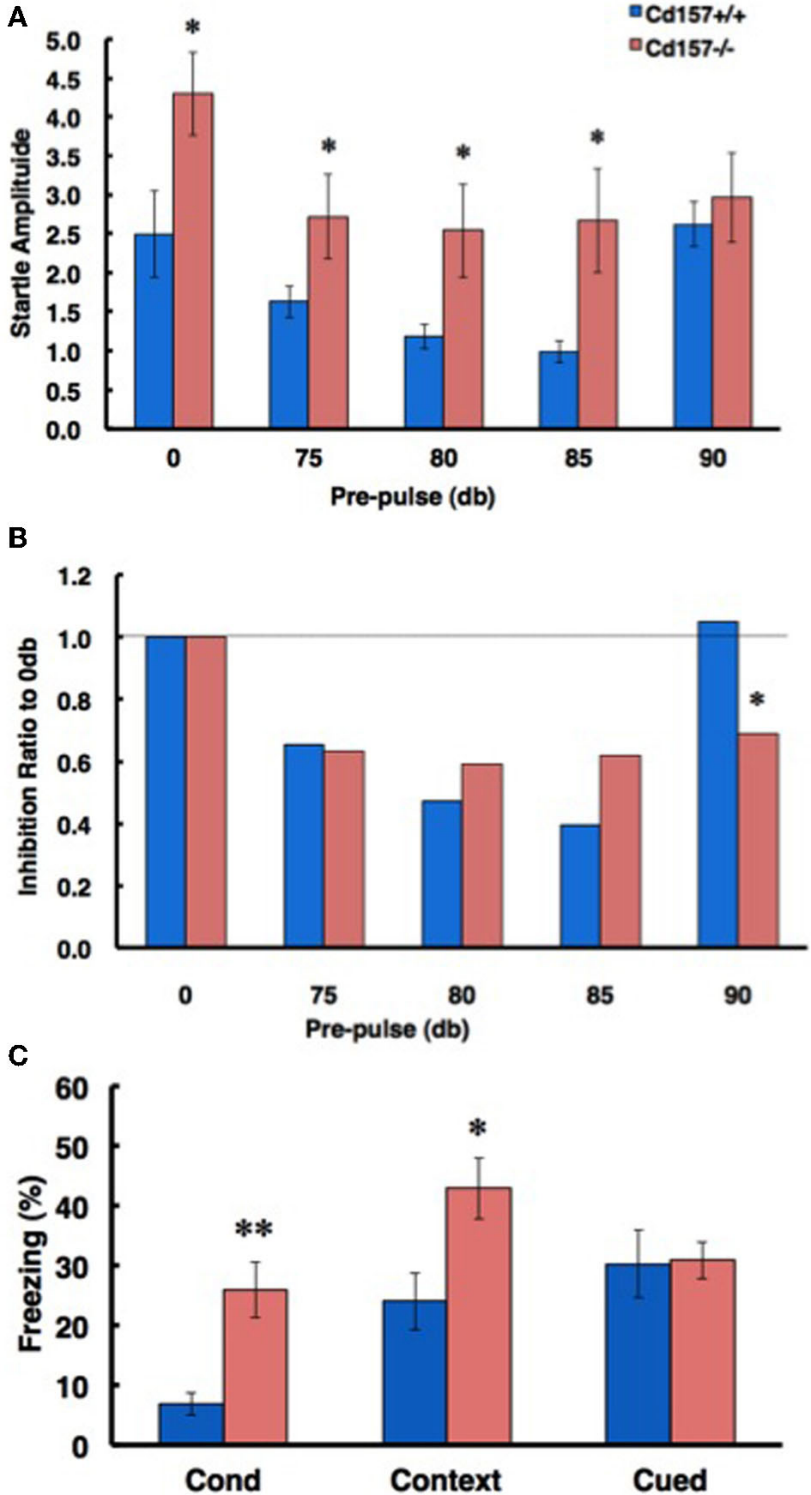
**Attention- and fear-related characteristics. (A)** In a pre-pulse inhibition paradigm based on the acoustic startle response, *CD157*^−/−^ mice exhibited an increased startle amplitude from 0-dB to 85-dB pre-pulse conditions (*n* = 10, ^*^P < 0.05). **(B)** The ratio of inhibition in *CD157*^−/−^ mice did not differ from that of the *CD157*^+/+^ mice up to the 85-dB condition, but the effect of inhibition was maintained with a 90-dB pre-pulse. Note that the 90-dB pre-pulse was normally ineffective in the *CD157*^+/+^ mice (*n* = 10, ^*^P < 0.05). **(C)** The ratio of freezing in *CD157*^−/−^mice was significantly increased in the conditioning and contextual test phases of the fear conditioning paradigm. The data are expressed as the means ± s.e.m. (*n* = 10, ^*^P < 0.05 or ^**^P < 0.01).

To confirm the sensory characteristics of the *CD157*^−/−^ mice, we measured their acoustic startle response. The startle response was significantly elevated in the knockout mice compared with that of the wild-type mice (0-db pre-pulse in Figure 10A; *n* = 10 in each genotype, *t* = 2.183, *P* < 0.05). The *CD157*^−/−^ mice exhibited greater startle responses under almost all pre-pulse conditions except for stimulation at 90 db (Figure 10B). In addition, sensory disturbance was also observed during a fear-conditioning paradigm (Figure 10C). The *CD157*^−/−^ mice exhibited a longer freezing time than did the *CD157*^+/+^ mice (*n* = 10 in each genotype; conditioning, *t* = 3.195, *p* < 0.01; contextual, *t* = 2.404, *P* < 0.05), indicating that the knockout mice have a disturbance in their attention or sensory gating.

### ADP-ribosyl cyclase activity

One important aspect addressed is that CD157 deficiency affected the metabolism of oxytocin (OT), a well-known hormone suspected to play a role in neuropsychiatric features (Higashida et al., 2012a,b; Lee, 2012). The authors have previously shown that deficiency in CD38 (another major member of the NADase/ADP-ribosyl cyclase family that shares 30% homology with CD157 (Malavasi et al., 2008; Lee, 2012) was associated with a dramatic decrease in plasmatic oxytocin leading to maternal and social behavior alterations (Jin et al., 2007; Higashida et al., 2012a,b).

**Figure 11:**
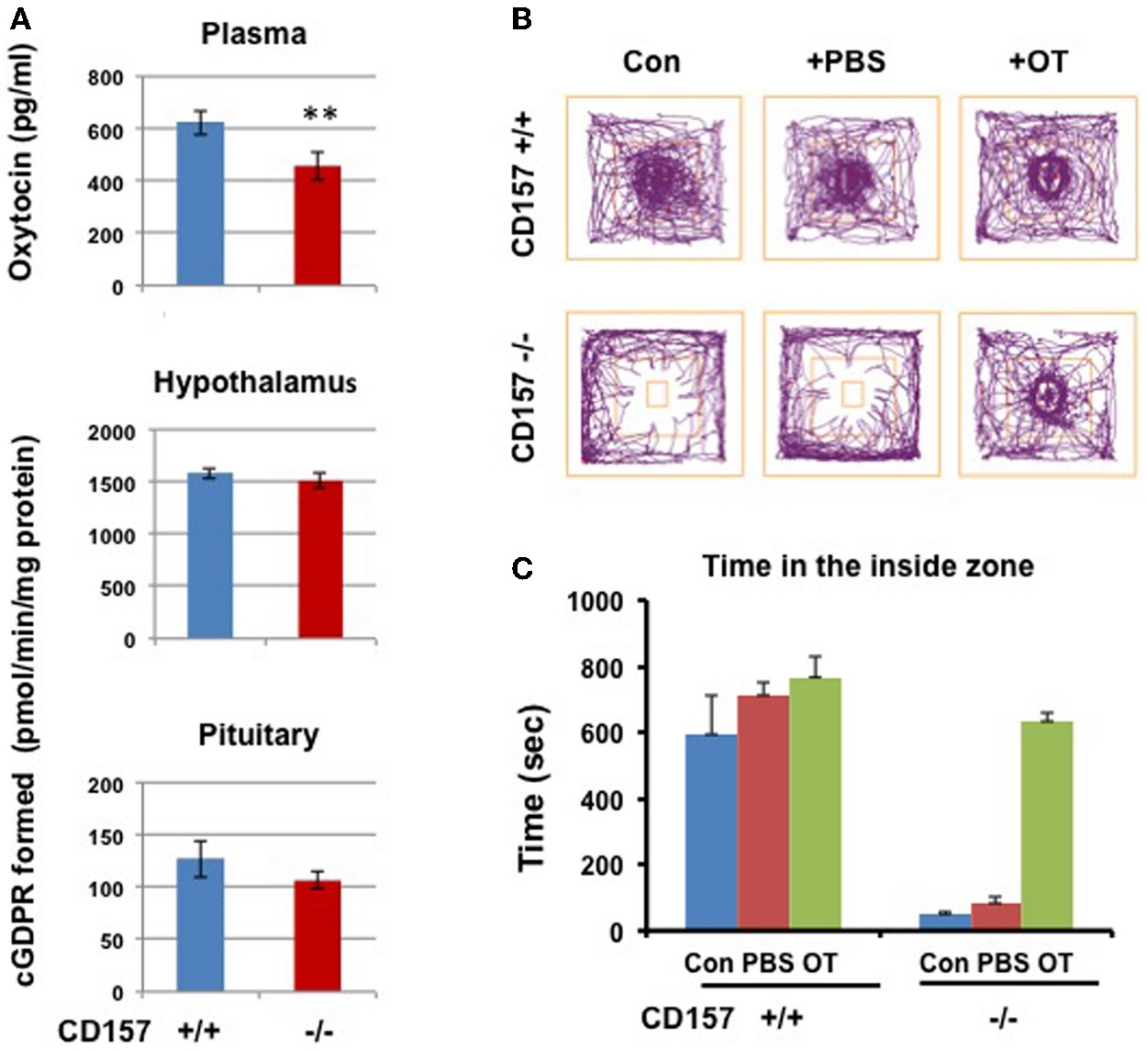
**ADP-ribosyl cyclase activity, oxytocin levels and social avoidance in the open field test. (A)** Plasma OT levels in *CD157*^+/+^ mice is higher than those in *CD157*^−/−^ mice (*n* = 6, ^**^P < 0.01, Student *t*-test). ADP-ribosyl cyclase activity measured with formation of cyclic GDP-ribose in the hypothalamus and pituitary in* CD157^+/+^* or *CD157*^−/−^mice (*n* = 6). **(B)** A schematic drawing of the social interaction test. The social target (a male mouse) was placed in the center immediately after a 10-min habituation. Representative track traces of *CD157*^+/+^ and *CD157*^−/−^ mice treated with or without PBS or OT (100 ng/kg of body weight, i.p.). **(C)** Time spent in the inside zone measured over 10 min in *CD157*^+/+^ and *CD157*^−/−^ mice treated with or without PBS or OT. The data are expressed as the means ± s.e.m. [*n* = 6–7, Two-Way ANOVA for genotypes and OT, *F*_(1, 11)_ = 13.01, *P* < 0.0001].

Therefore, we examined ADP-ribosyl cyclase activity (Figure 11). There were no significant differences in ADP-ribosyl cyclase activity between the two genotypes in the hypothalamus (1584.1 ± 53.1 for CD157^+/+^ mice vs. 1509.2 ± 71.5 pmol/min/mg protein for *CD157*^−/−^ mice) and pituitary (126.7 ± 17.2 vs. 106.3 ± 8.7 pmol/min/mg protein for *CD157*^+/+^ and *CD157*^−/−^ mice, respectively; Figure 11A).

We measured the plasma OT levels in both genotypes. The plasma concentration of OT in *CD157*^−/−^ mice (457.1 ± 54.1 pg/ml, *n* = 6) was significantly lower than that in *CD157*^+/+^ mice (623.5 ± 46.1 pg/ml, *n* = 6, *P* < 0.01; Figure 11A).

### Recovery of sociability by oxytocin

Based on our data from plasma OT levels, we tested the effects of OT on impaired emotional behavior in *CD157*^−/−^ mice. Here, we used the newly reported simple two-step social interaction test in the open field (Chaudhury et al., 2013). As shown in Figures 11B,C, a clear social avoidance was detected in *CD157*^−/−^ mice, compared to *CD157*^+/+^ mice [*n* = 6–7, Two-Way ANOVA for genotypes, *F*_(1, 11)_ = 94.91, *P* < 0.0001]. *CD157*^+/+^ mice showed significantly higher interactions than that in *CD157*^−/−^ mice with subcutaneous injection of PBS [*n* = 6–7, Two-Way ANOVA for PBS, *F*_(1, 11)_ = 28.59, *P* < 0.0001]. At 20 min from intraperitoneal injections of OT (100 ng/Kg of body weight), the *CD157*^−/−^ mice recovered to a level identical to that in *CD157*^+/+^ mice [*n* = 6–7, Two-Way ANOVA for genotypes and OT, *F*_(1, 11)_ = 13.01, *P* < 0.0001].

### CD157 in the amygdala

**Figure 12:**
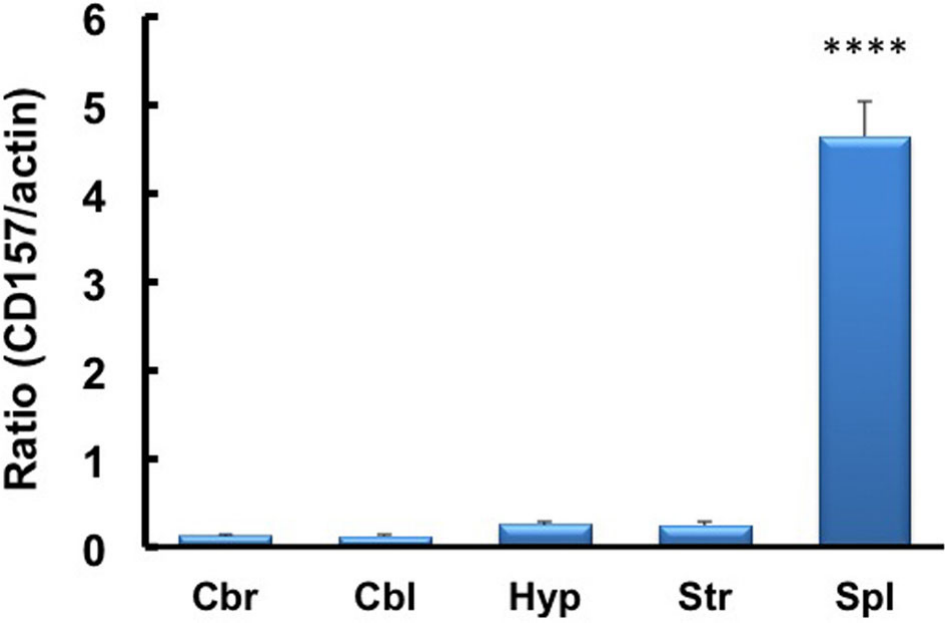
**RT-PCR analysis of *CD157* mRNA expression levels in the four brain regions and spleens of mice using β-actin mRNA as an internal control**. The quantitative data are expressed as the means ± s.e.m. (*n* = 5 independent experiments of *CD157*/actin). ^****^P < 0.001 from the striatum, Student *t*-test. Abbreviations used: Cbr, cerebrum; Cbl, cerebellum; Hyp, hypothalamus; Str, striatum; Spl, spleen.

**Figure 13:**
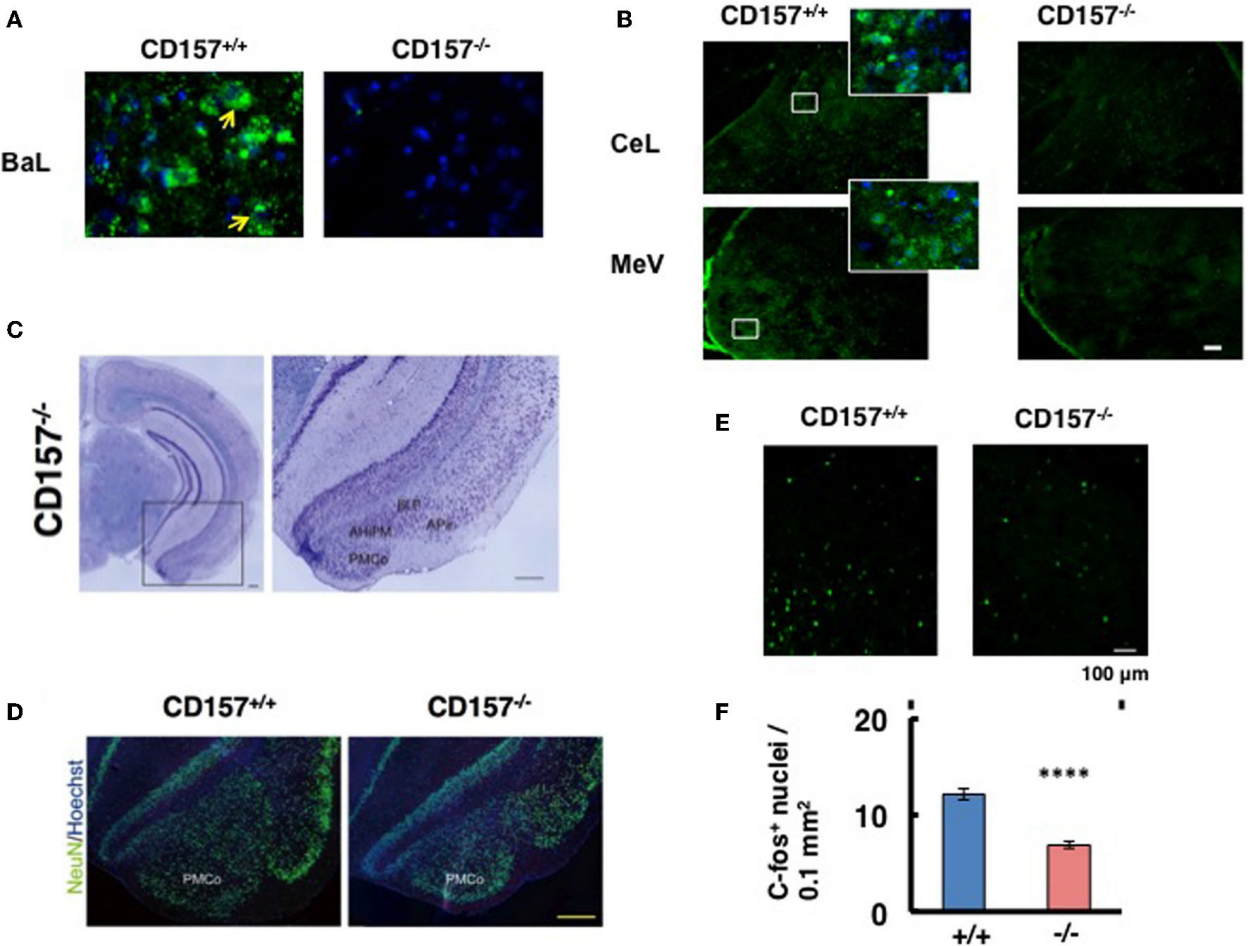
**CD157 expression in the amygdala**. **(A,B)** Representative photos display CD157 immunoreactivity (green, indicated by arrows) in the basolateral amygdaloid nucleus anterior part (BaL), or central amygdaloid nucleus (CeL) and medial amygdaloid nucleus posteroventral part (MeV) of adult male * CD157^+/+^* or *CD157*^−/−^ mice. Hoechst-stained nuclei are shown in blue. The insets are enlarged areas indicated in lower-magnification photos. The scale bar represents 100 μm. **(C)** Nissl-stained coronal sections of *CD157*^−/−^ mice (anteroposterior position at Bregma −3.08 mm). Magnified images of the amygdala, marked with a black square, is shown to the right. The amygdala, including the amygdalohippocampal area posteromedial part (AhiPM), the posterior part of the basolateral amygdaloid nucleus (BLP), the amygdalopiriform transition area (APir) and the posteromedial cortical amygdaloid nucleus (PMCo) are indicated. Scale bar: 200 μm. **(D)** NeuN-immunostained cells (neurons) are shown in green, and Hoechst-stained nuclei are shown in blue in * CD157^+/+^* or *CD157*^−/−^ mice. Scale bar: 200 μm. **(E)** c-Fos immunostaining in the amygdala in mice exposed to the new environment in the open field test for 10 min. Photomicrographs of coronal sections showing c-Fos immunoreactivity as Alexa 488 fluorescence. **(F)** The number of c-Fos-immunoreactive cells in the amygdala in *CD157*^+/+^ or *CD157*^−/−^ mice as in **(E)**. The data are expressed as the means ± s.e.m. (*n* = 4, ^****^P < 0.001).

We investigated the neurological cues underlying the anxiety-related and depression-like behaviors in *CD157*^−/−^ mice at the tissue and molecular levels. First, we examined the protein expression of CD157 in the adult brains of wild-type males using a specific antiserum developed against CD157 (see Methods). Consistent with the low level of *CD157* mRNA expression in the brain regions compared with the expression level in the spleens of wild-type adults (Figure 12), weak but distinct immunoreactivities were detected in the amygdala: the basolateral amygdaloid nucleus anterior region, the central amygdaloid nucleus and the medial amygdaloid nucleus posteroventral region in *CD157*^+/+^ mice, but not in *CD157*^−/−^ mice (Figures 13A,B). In other brain regions, little or no immunoreactivity was detected (data not shown).

Based on these observations, we macroscopically examined the brains of these mice. Macroscopic abnormalities were not obvious, and there was no difference in the wet weights of the brains in the two genotypes (*n* = 20, 0.43 ± 0.4 g vs. 0.43 ± 0.2 g for wild-type and knockout mice, respectively). Subsequently, we microscopically observed the brains. Nissl staining was performed on male brains, and no or less structural abnormalities were observed in the regions of the brain tested, including the cortex and the hippocampus, in *CD157*^−/−^ mice (Figure 13C, left panel). However, the amygdala regions seem to be altered in *CD157*^−/−^ mice, including the amygdalohippocampal area posteromedial part (AhiPM), the posterior part of the basolateral amygdaloid nucleus (BLP), the amygdalopiriform transition area (APir) and the posteromedial cortical amygdaloid nucleus (PMCo; Figure 13C, right panel). Then, we compared the amygdala between two genotypes by neuron-specific NeuN staining (Figure 13D). The amydgala regions in the *CD157*^−/−^ mice seem to be smaller compared with those of the *CD157*^+/+^ mice. However, when the number of DAPI-stained nuclei was measured in the PMCo, the cell density was approximately equal in both genotypes: 193.3 ± 5.2 cells/μm^2^ for *CD157*^+/+^ mice and 204.5 ± 6.0 cells/μm^2^ for *CD157*^−/−^ mice (*t* = 0.15, *n* = 5).

To gain a functional change of the amygdala in response to environmental stimuli, we quantified the amount of c-Fos immunoreactivity in the amygdala of mice from both genotypes (Figure 13E). When the mice were exposed to the new environment in the open field test for 10 min, c-Fos immunoreactive cell number was less evident in *CD157*^−/−^ mice than in *CD157*^+/+^ mice (*n* = 4, *t* = 7.212, *P* < 0.001; Figure 13F). These results suggest that the amygdala is seemingly less developed and neurons activated in the amygdala is impaired in the knockout mice.

## Discussion

Our results show that young adult male mice of 8- to 10-weeks old with the *CD157* (*BST1*) null mutation display robust and well-replicated emotion-related behavioral phenotypes: *CD157*^−/−^ mice displayed severe anxiety-related behaviors for the novel environment; *CD157*^−/−^ mice also exhibited anxiety for non-social and/or social novel targets; Weak sociability with novel target mice and social avoidance for target males were also prominent phenotypes. In addition, *CD157*^−/−^ mice displayed depression-like behaviors. All of these social behavior impairments in multiple paradigms seem to suggest psychiatric traits in *CD157*^−/−^ mice, because the multiple tasks applied here are well-established tools for use with animal models of human psychiatric disorders (Crawley, 2008).

The anxiety-related behavioral phenotype detected by the standard test for anxiety was fully reversed by diazepam, a typical anti-anxiety drug, and the recently developed anti-depressant drug, mirtazapine. Depression-like behaviors detected by the tail suspension paradigm were reversed by diazepam, and mirtazapine-treated animals recovered to a greater degree than the control animals. The same phenotypic deficiencies seen in the forced swimming test were not recovered by diazepam, but were effectively rescued by mirtazapine. The phenotype of climbing to the wall, which may also represent some sensory deficit and be consistent with the altered state of fear observed in *CD157*^−/−^ mice (Figure 10), was markedly rescued by both drugs. Such pharmacological intervention effectiveness suggests a robust signal-to-noise disturbance in emotional phenotypes and provides an easy replicable baseline for the therapeutic response in *CD157*^−/−^ mice. Although the data show slight sensitivity differences of drugs on both phenotypes, because we used a single way of application and dose of drugs (Figures 7, 8), it is too early to determine the detailed pharmacological features of emotional phenotypes in the knockout mice. Genetic rescue of the *CD157*^−/−^ mice was admittedly not provided here, likely because of comprehensible difficulties in infecting and expressing the glycosylphosphatidylinisotol-linked molecule. Genetic manipulations for rescue of phenotypes, such as brain re-expressing of CD157 with the lenti-virus infection technique (Jin et al., 2007; Akther et al., 2013), would be more confirmative.

We did not detect any apparent physical disability in young adult *CD157*^−/−^ mice under less influence of aging. When the knockout mice were forced to move during foot printing or on the rota rod, no apparent abnormality in coordinate motion or learning and memory for the movement was detected. In contrast, *CD157*^−/−^mice generally displayed less voluntary activities during daily life with 24-h monitoring for 7 days in their home cage, compared to home cages of wild-type mice (Figure 4). This striking discrepancy in forced and voluntary movements, to our knowledge, may be a characteristic behavioral measurement that revealed a robust emotion-related disability in knockout mice. Though current home cage recordings by the sensor beam did not reveal how the mice behaved exactly, we estimated that they squatted down in a single spot for long time, likely as a result of an apathy state derived from depressive emotion in the *CD157*^−/−^ mice. The precise patterns of individual behavior should be validated. On the other hand, interestingly, the day and night cycle was the same as for control mice, indicating that the circadian rhythm is likely not affected in *CD157*^−/−^ mice.

Interestingly, several genome-wide association and meta-analysis studies for PD have identified intronic single-nucleotide polymorphisms (SNPs) in the *CD157* (also known as *BST1*) gene on human chromosome 4p15 as new susceptibility loci (Nalls et al., 2011; Lill et al., 2012; Noyce et al., 2012), although one may argue that the *CD157* common SNPs provide not large risk. Additional genetic and environmental factors may thus be needed to unravel a pathogenic role of CD157 genetic modification or deletion (Chen et al., 2013).

The neuropsychiatric comorbidities in PD have not intensively been studied in an appropriate animal model (Dawson et al., 2010; Campos et al., 2013), although anxiety has been observed in conventional neurotoxin-treated rats and mice and in Parkin-deficient mice (Branchi et al., 2008; Dawson et al., 2010; Rane et al., 2012; Campos et al., 2013). It is of interest to test if these behavioral impairments in *CD157*^−/−^ mice likely reflect impairment of the amygdala (Harding et al., 2002; Surdhar et al., 2012). However, there is no direct evidence that CD157 plays a role in neuronal migration during neurogenesis, although CD157 binds integrins in human monocytes (Lo Buono et al., 2011, 2014) and plays a role in neutrophil migration (Quarona et al., 2013). Our preliminary results show that CD157 is expressed in nestin-positive neural stem cells near the brain ventricular zone. Therefore, it is likely that a CD157 deficiency might cause amygdala's abnormalities.

Recently, it has been estimated that abnormalities in additional neuronal regions rather than the nigrostrial region are potentially involved in PD progression and may contribute to the appearance of non-motor symptoms, such as anxiety, depression, olfactory and memory impairment, sleep abnormalities and gastrointestinal disturbances (Tadaiesky et al., 2008; Wattendorf et al., 2009; Starkstein et al., 2013; Storch et al., 2013). Anxiety and depression are the earliest PD manifestations, and patients with high anxiety are at an increased risk for PD (Richard, 2005; Drijgers et al., 2010; Schrag and Leentjens, 2012). The underlying biological mechanisms that lead to these symptoms during any stage of the disease, including the pre-motor phase, are unknown (Prediger et al., 2012; Storch et al., 2013). Therefore, it is reasonable that our current experiments were designed to examine emotion-related phenotypes in the time window of 8- to 10-weeks-old. Further validation is necessary to determine a relationship between emotion-related phenotypes in *CD157* knockout mice and any small aspects of human emotion phenotypes in PD.

At present, we cannot explain the discrepancy between plasma and hypothalamic levels of OT (Figure 11). As the majority of OT neurons release OT into the blood, we speculate that the release of OT from axon terminals in the pituitary may be attenuated in *CD157* knockout mice without detectable changes in OT contents in the hypothalamic nuclei, which are enriched in OT. It is clear that OT neurons project concomitantly to the posterior pituitary and brain regions controlling fear responses, such as the central amygdala (Knobloch et al., 2012; Knobloch and Grinevich, 2014). Therefore, CD157 may directly or indirectly affect the central axonal release of OT. The observed alteration of the OT system (plasma concentrations) and compensatory effects of OT on behaviors provide clinically and therapeutically relevant insights.

An alternative scenario underlying the contribution of OT in *CD157* knockout mice is disruption in OT signaling in the brain. To investigate this issue, data regarding expression of OT receptors in the relevant brain areas are required. In a preliminary experiment, we measured the OT receptor mRNA levels in the amygdala (*n* = 5). Unfortunately, we failed to detect any differences in OT receptor mRNA level in either genotype. Further detailed analyses are required to determine the mRNA and protein levels of molecules related to OT signaling in each subregion of the amygdala.

There is controversy regarding whether peripherally administered OT can efficiently penetrate the blood–brain barrier (Churchland and Winkielman, 2012; Ludwig et al., 2013). Therefore, the precise mechanism underlying the action of OT in the present study remains unclear. However, numerous studies in rodents and humans have shown convincing pro-social effects of OT applied peripherally, including intranasal application (Neumann et al., 2013; Striepens et al., 2013; Watanabe et al., 2014).

In conclusion, we have shown that CD157 plays a role in the brain. Taken together with previous reports (Ishihara and Hirano, 2000; Yilmaz et al., 2012; Quarona et al., 2013), these observations indicat that CD157 has multiple functions in a variety of tissues. The present findings support the novel suggestion that CD157 can be defined as *a neuro-entero-immunological regulator*, which is important when considering the biological significance of the products, including cyclic ADPR, of the CD38 family. The lack of differences in ADP-ribosyl cyclase activities in two distinct areas of the brain observed in the present study may be the result of a reciprocal surrogate role played by other members of the same family. Therefore, further experiments using double *CD38/CD157* knokout mice are required to address several of the remaining open questions. Future studies may include validation to support the link between the intronic common *CD157* SNPs and the emotion phenotypes relevant to anxiety or depression observed during the premotor phase that precedes classical motor features in PD patients.

## Author contributions

Haruhiro Higashida, Olga Lopatina, Toru Yoshihara, Tomoko Nishimura, Jing Zhong, Shirin Akther, Osamu Hori, Kohei Sumi, Makoto Sato, and Katsuhiko Ishihara designed the experiments. All authors performed the experiments. Olga Lopatina, Toru Yoshihara, and Tomoko Nishimura conducted behavioral experiments as independent group leaders. Haruhiro Higashida, Olga Lopatina, Toru Yoshihara, and Katsuhiko Ishihara wrote the manuscript.

### Conflict of interest statement

The authors declare that the research was conducted in the absence of any commercial or financial relationships that could be construed as a potential conflict of interest.
